# Selection and evaluation of new reference genes for RT-qPCR analysis in *Epinephelus akaara* based on transcriptome data

**DOI:** 10.1371/journal.pone.0171646

**Published:** 2017-02-09

**Authors:** Huan Wang, Xiang Zhang, Qiaohong Liu, Xiaochun Liu, Shaoxiong Ding

**Affiliations:** 1 State Key Laboratory of Marine Environmental Science, College of Ocean and Earth Sciences, Xiamen University, Xiamen, Fujian, China; 2 State-Province Joint Engineering Laboratory of Marine Bioproducts and Technology, College of Ocean and Earth Sciences, Xiamen University, Xiamen, Fujian, China; 3 State Key Laboratory of Biocontrol, Institute of Aquatic Economic Animals and Guangdong Provincial Key Laboratory for Aquatic Economic Animals, School of Life Sciences, Sun Yat-Sen University, Guangzhou, Guangdong, China; Northwestern University Feinberg School of Medicine, UNITED STATES

## Abstract

Groupers are an economically important fish species in world fishery markets. Because many studies using RT-qPCR have addressed gene expression in groupers, appropriate reference genes are required to obtain reliable and accurate results. In this study, the most suitable reference genes were identified from eleven candidate genes of one of the most valuable species, *Epinephelus akaara*, in a range of different experimental conditions. Using the software packages geNorm, NormFinder, BestKeeper and refFinder, three traditionally used reference genes, *β*-actin (*β-ACT*), glyceraldehyde-3-phosphate dehydrogenase (*GAPDH*), and *beta-2-microglobulin* (*B2M*), were identified as not suitable for *E*. *akaara* gene expression studies, whereas two newly identified reference genes, *conserved oligomeric Golgi complex subunit 5* (*Cog5*) and *brefeldin a-inhibited guanine nucleotide-exchange protein 1* (*ARFGEF1*), could be universally applied under all the tested conditions. These data provide the foundation for more precise results in RT-qPCR studies of gene expression in *E*. *akaara* and other Epinephelus species.

## Introduction

Currently, real-time quantitative reverse transcription polymerase chain reaction (RT-qPCR) is a ubiquitous mainstay technology for quantifying gene expression because of its advantages, which include accuracy, specificity, sensitivity, reproducibility and convenience [1, 2]. Despite the numerous advantages, normalization to a constitutively expressed gene is required to avoid experimental errors caused by different sample amounts, variations in RNA, enzymatic efficiency for cDNA transcription and PCR efficiency [3, 4]. Traditionally, the reference genes that have been used are primarily housekeeping genes, such as *β*-actin (*β-ACT*), glyceraldehyde-3-phosphate dehydrogenase (*GAPDH*), tubulin (*TUB*), elongation factor 1-α (*EF1*α), polyubiquitin (*UBQ*) and ribosomal RNAs (18S or 28S rRNA). However, many studies show that the transcript levels of these genes vary considerably across cellular conditions [5–9], which indicate that they should not be commonly used in all gene expression analysis situations. Therefore, the selection of a suitable reference gene to study specific objects or experimental conditions is an important factor for gene expression analysis by RT-qPCR. Recently, with the rapid development of new biotechnology, transcriptome sequencing has been applied to identify new reference genes in various species, including monkey [10], buckwheat [11], crazyweed [12], deer [13], rice [14], and oilseed rape [15], and the results show that the new reference genes are more stably expressed and that the orthologs of these genes are suitable reference genes in different species that have a close relationship [11]. Therefore, examination of transcriptome-sequencing data may be a useful method to identify more reliable reference genes for RT-qPCR studies on specific fauna or under different experimental conditions.

Grouper (Perciformes: Epinephelidae) have high economic value in Asian fishery markets, and grouper mariculture has rapidly developed due to the insufficient supply of wild caught fish, which does not satisfy the high demand of markets [16]. However, similar to other aquaculture species, the grouper aquaculture industry is also hindered by several problems that remain unresolved. One constraint of grouper culture is the production of fertilized eggs due to the lack of a standardized method for controlling sex change and the unavailability of mature male brood stock caused by the protogynous hermaphrodite mode of reproduction [17]. Larval production is another challenge for grouper farming because of the high level of mortality of embryos and larvae, which may be associated with biological aspects, nutritional deficiencies, genetic makeup of the parent population, variations in ecological factors and outbreaks of infectious disease [18, 19]. The variation in ecological factors and the diseases caused by pathogens are also large problems for farming grouper to commercial size. Salinity is an important ecological factor and not only influences the egg fertilization and incubation, early embryogenesis and larval growth but is also a key factor in controlling growth [20, 21]. *Vibrio alginolyticus*, a causative agent of outbreaks of vibriosis, is one of the most serious diseases and a common problem during various stages of grouper culture that causes fish mortality and serious economic losses [22, 23]. The natural disease caused by *V*. *alginolyticus* in fish includes symptoms of septicaemia, haemorrhage, dark skin, and ulcers on the skin surface in some cases [24]. In recent years, to contribute to the development of artificial grouper breeding and the protection of wild resources, an increasing number of investigations use RT-qPCR to examine the functional genes involved in the aspects discussed above. However, in the gonadal transcriptome data of an important grouper species, *Epinephelus akaara*, we found variable expression levels of some commonly used reference genes, such as *β-ACT*. Therefore, these genes may not be suitable as reference genes for analyses of gene expression in grouper and could lead to the erroneous normalization of RT-qPCR data. Thus, the identification of suitable reference genes in grouper studies is crucial for the accurate profiling of gene expression.

The objective of this study was to evaluate the appropriateness of traditional reference genes and new candidate reference genes derived from our previous transcriptome sequencing data of *E*. *akaara* and identify the most suitable reference genes for different experimental conditions. To the best of our knowledge, this report is the first assessment of valid reference genes for RT-qPCR studies on groupers. Because the Epinephelus species has the characteristics of a relatively short evolutionary time and closely related phylogeny [25], the identification of these new reference genes for the normalization of RT-qPCR data not only will be an essential tool for future gene expression studies in *E*. *akaara* but also may be applicable to other commercially important Epinephelus species, such as *E*. *coioides*, *E*. *lanceolatus*, *Cromileptes altivelis* and *Anyperodon leucogrammicus*.

## Materials and methods

### Sample preparation and experimental procedures

All *E*. *akaara* used in this study were obtained from a local commercial aquatic farm in Fujian Province, China. This study was conducted in strict accordance with the guidelines for the Care and Use of Laboratory Animals. The Institutional Animal Care and Use Committee of Xiamen University approved the protocol. All fish surgery procedures were conducted under MS-222 (Tricaine Methanesulfonate) to induce sedation and anaesthesia. All eggs, larvae, and tissue samples were incubated in an RNA fixer (Aidlab, Beijing, China) and stored at -80°C for RNA isolation.

All experiments were performed according to the Minimum Information for Publication of Quantitative Real-Time PCR Experiments (MIQE) guidelines [26]. A detailed description of the sample preparation and experimental procedures, including information on sample collection under different experimental conditions, RNA isolation, cDNA synthesis, primer design, stand curve analysis, and RT-qPCR amplification conditions, is found in the S1 File.

### Selection of the candidate reference genes

For the selection of new candidate reference genes, we analysed our previous gonadal transcriptome sequencing data (PRJNA277894) from the following five different gonadal development phases of *E*. *akaara*: undifferentiated-phase (UN), developing female (DF), developing male (DM), mature female (MF) and mature male (MM) [27]. To estimate the expression stability of each gene, we analysed the raw data for all genes as follows: (1) sorting the genes expressed in all of the tested tissues; (2) calculating the mean expression values (MVs), standard deviations (SDs) and coefficients of variation (CVs); (3) screening the genes that satisfied the conditions described by de Jonge et al [28]; and (4) ordering the gene list according to the lowest CV value.

Based on these selection procedures for the transcriptome sequencing data, 10 genes that had a minor variation in expression were selected. Of these genes, 8 were newly identified candidate reference genes, including *brefeldin a-inhibited guanine nucleotide-exchange protein 1* (*ARFGEF1*), *conserved oligomeric Golgi complex subunit 5* (*Cog5*), *putative ATP-dependent RNA helicase DHX30* (*DHX30*), *neuron navigator 3* (*Nav3*), *homeodomain-interacting protein kinase 3* (*Hipk3*), *probable E3 ubiquitin-protein ligase MYCBP2* (*Mycbp2*), *E3 ubiquitin-protein ligase MGRN1* (*Mgrn1*), and *60S ribosomal protein L17* (*RPL17*), and two were traditionally used reference genes, *GAPDH* and *beta-2-microglobulin* (*B2M*). The most commonly used reference gene, *β-ACT*, in grouper was also evaluated in our study.

### Primer specificity and RT-qPCR efficiency analysis

We used full-length unigene sequences from gonadal transcriptome data to design specific primers for RT-qPCR. The RT-qPCR products ranged from 86 to 243 bp. The specificities of all the primers were demonstrated by the single bands of expected size in agarose gel electrophoresis and by the single-peak melting curves of the RT-qPCR products (S1 Fig). The Sanger sequencing of amplicons with alignment confirmed the specificity and accuracy of the designed primers. The Cq values of all the primers were greater than 35 in the non-template controls (NTC). Therefore, minute amounts of primer dimers did not affect the fluorescence level of the amplified target gene. The amplifications detected in non-reverse transcription controls (NRT) were similar to the NTCs, which indicated that there was no genomic DNA contamination. The E-values of the eleven genes in this study were between 91% and 99%, which were within the acceptable range. The primer sequences and relevant amplification information are presented in Table 1.

**Table 1:** Eleven selected candidate reference genes, gene function, primers and different parameters derived from RT-qPCR data analysis.

**Gene**	**Function**	**Primer Forward(F)/Reverse(R)**	**Amplicon size (bp)**	**Efficiency (%)**	**R**^**2**^	**NTC(Cq)**	**NRT(Cq)**
β-ACT	Cytoskeletal structural protein	1. F:GCGACCTCACAGACTACCT 2. F: CTGGGCAACGGAACCT	228	94	0.991	36.59	35.78
GAPDH	Oxidoreductase in glycolysis and gluconeogenesis	3. F: TTGATGTCGTCGTATTTGG 4. F: CTGCCTCTACTGGTGCTG	154	99	0.994	36.47	36.15
RPL17	Structural component of 60S ribosomal subunit	5. F: ACACTGGTGCTTGACGATG 6. F: CCGAGAACCCGACTAAAT	149	96	0.997	35.71	35.92
B2M	Beta-chain of major histocompatibility complex class I	7. F: GCTTACATATCTGATTCCCACTC 8. F: CCAACCCAACACCCTGA	243	92	0.997	36.13	35.64
ARFGEF1	ADP-ribosylation factor-1	9. F: AACAAGTTGGGCTTAGATT 10. F: CAGGCTCAGAACAGGGTA	169	98	0.991	37.02	36.80
Cog5	Subunit of large Golgi-associated protein complex	11. F: TTCAGGATTGGCGTCTT 12. F: GGAGCGTGATGATTATGG	143	97	0.992	35.85	35.25
DHX30	ATP-dependent RNA helicase	13. F: CAGCACGGCTCTAATGAA 14. F: CCTCGTCTGGGCAAAGT	192	93	0.993	37.82	36.93
Nav3	Neurone navigator protein	15. F: TATGACTTCGCCCGTTTG 16. F: TGCTCTTTCTGCCCATCA	94	97	0.995	39.22	38.77
Hipk3	Homeodomain-interacting protein kinase	17. F: CGTTACAGTGCCGAGTTT 18. F: ACAGGCGGTAATAGAGTAGAT	131	92	0.993	36.78	36.43
Mycbp2	E3 ubiquitin ligase	19. F: CAGAGGTGCGTCCAAGAG 20. F: AGGTGACAGGGTAAGGGTG	117	91	0.996	35.17	35.71
Mgrn1	E3 ubiquitin ligase	21. F: TCGGCAACCTTTGATTC 1. F: CAAGTGGTGGATGGAGTG	86	95	0.995	36.71	36.03

### Gene stability analysis

The PCR data were analysed using three approaches, geNorm [4], NormFinder [29] and BestKeeper [30]. For all three programs, Ct values were transformed into Cq values by the standard formula *Cq = Log (2)/Log (E)*, where E is the efficiency of the amplification of each primer pair. Then, for both geNorm and NormFinder, the Cq values were transformed to relative quantities using the delta-Cq formula *Q = 2* ^delta-Cq^, where delta-Cq is the difference between the sample with the lowest Cq and the Cq value of the sample in question. The geNorm program calculates gene expression stability (M-value), which is based on the pairwise variation of a single reference candidate gene relative to all other tested control genes. Genes with an M-value below the threshold of 1.5 were those stably expressed and lower M-values indicated greater stability of a gene. The geNorm program also estimates the pairwise variation (V_n_/V_n+1_) between two sequential normalization factors, NF_n_ and NF_n+1_, to determine the optimal number of reference genes required. The optimal number of reference genes is necessary to satisfy V_n_/V_n+1_ below the cut-off value of 0.15. NormFinder uses an ANOVA-based model to identify the most stable reference genes. NormFinder calculates intra- and inter-group variations, and genes with the least variations are those that the most stable. BestKeeper calculates the SD and CV based on the Cq values of each reference gene. Genes with an SD value of < 1.0 are stable, and the gene with the lowest SD and CV values was identified as the most stable. Additionally, refFinder, a user-friendly comprehensive tool, was used to integrate the results from the three programs and select the best reference gene.

### Validation of the recommended reference genes

To validate the stability of the identified reference genes, the expression levels of two target genes were analysed in different experimental conditions. A non-mammalian myostatin, *growth and differentiation factor-8* (*MSTN*), was used to validate the expression levels of the most stable, the universal, and the least stable reference genes for *E*. *akaara* during gonad development and early ontogenetic development phases. An important immune responsive cytokine, *interleukin* (*IL*)*-1β*, was used to validate the expression levels of the most stable, the universal, and the least stable reference genes for salinity treatment and *V*. *alginolyticus* challenged *E*. *akaara*. Relative quantification of these two genes in different samples was performed using the 2^-ΔΔCt^ method [31].

## Results

### Expression stability of candidate reference genes during different gonad development phases of *E*. *akaara*

**Fig 1:**
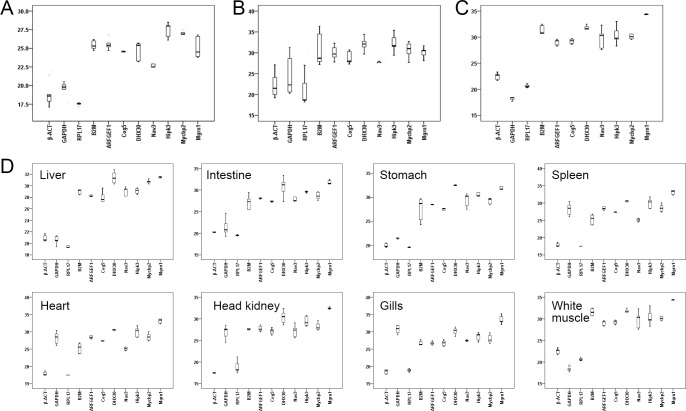
Expression levels of the 11 candidate reference genes. Graphical representation of absolute Cq values for each gene analysed during different gonad development phases (A), during early ontogenetic development stages (B), for the salinity treatment of *E*.*akaara* (C), and for *V*.*alginolytics* challenged *E*.*akaara* (D). Values are Cq values. A line across the box depicts the median. The box indicates the 25th and 75th percentiles. Whiskers represent the maximum and minimum values.

**Fig 2:**
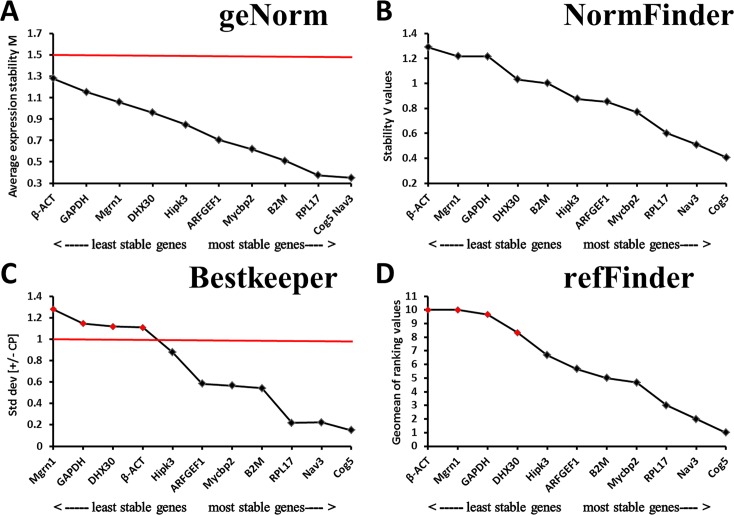
Determination of the expression stability during gonad development phases according to different programs. (A) geNorm, the red line indicates the geNorm cut-off value of 1.5. (B) NormFinder. (C) BestKeeper, the red line indicates the BestKeeper cut-off value of 1.0. (D) refFinder, the red indicates the gene expression was unstable.

**Fig 3:**
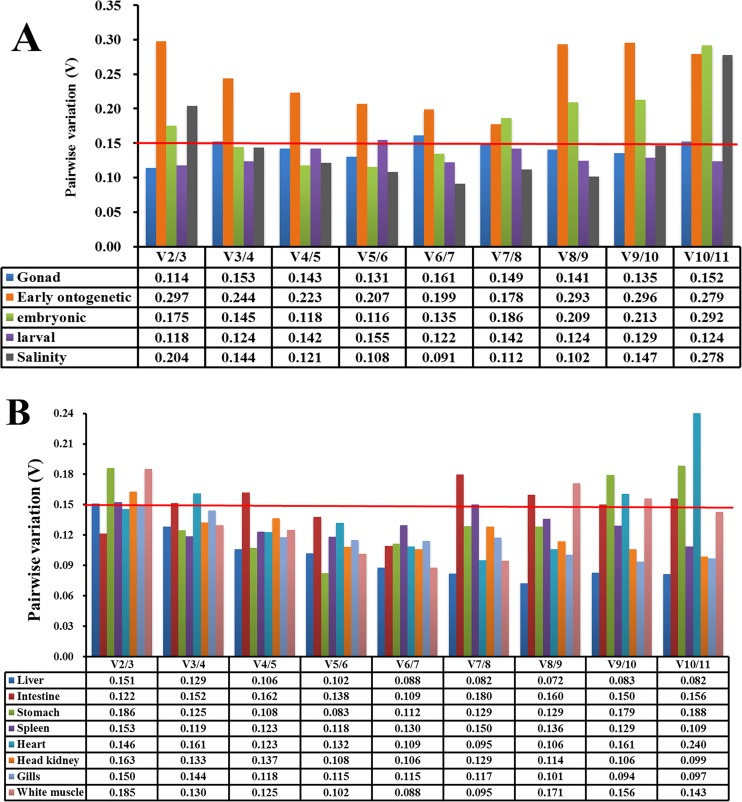
Determination of the optimal number of reference genes. Pairwise variations (V) of the 11 candidate reference genes were calculated by geNorm to determine the optimal number of reference genes for the accurate normalization during the gonad development phases, during the entire early ontogenetic development stage and in the separate embryonic ontogenesis stage and larval ontogenesis stage, and for salinity treatment samples (A) and in different tissues after *V*. *alginolyticus* challenge (B). The black line indicates the geNorm cut-off value of 0.15.

As shown in S1 Table and Fig 1A, the Cq values of the eleven candidate reference genes during *E*. *akaara* gonad development ranged between 16.0 and 30.5. *RPL17* was the most highly expressed gene, with Cq values ranging between 16.0 and 17.7, followed by *β-ACT* and *GAPDH*, which exhibited Cq values from 17.9 to 21.7 and from 17.1 to 21.8, respectively. The remaining genes had Cq values greater than 22, with the highest Cq value (30.5) exhibited by *Hipk3*. For expression stability analysis, the results from the geNorm, NormFinder, and BestKeeper programs were combined, and seven genes, *Hipk3*, *ARFGEF1*, *B2M*, *Mycbp2*, *RPL17*, *Nav3*, and *Cog5*, were stably expressed during different gonad development phases of *E*. *akaara* (Fig 2A, 2B, and 2C). The overall ranking order of stability of candidate reference genes determined by refFinder was *Cog5*>*Nav3*>*RPL17*>*Mycbp2*>*B2M*>*ARFGEF1*>*Hipk3*>*DHX30*>*GAPDH*>*Mgrn1*>*β-ACT* (Fig 2D). The results of the pairwise variation calculation showed that the V_2/3_ value was less than 0.15, which indicated that two reference genes were required for normalization (Fig 3A). Thus, we recommend *Cog5*/*Nav3* as the best reference gene pair for the normalization of *E*. *akaara* gonad development.

### Expression stability of candidate reference genes during *E*. *akaara* early ontogenetic development stage

**Fig 4:**
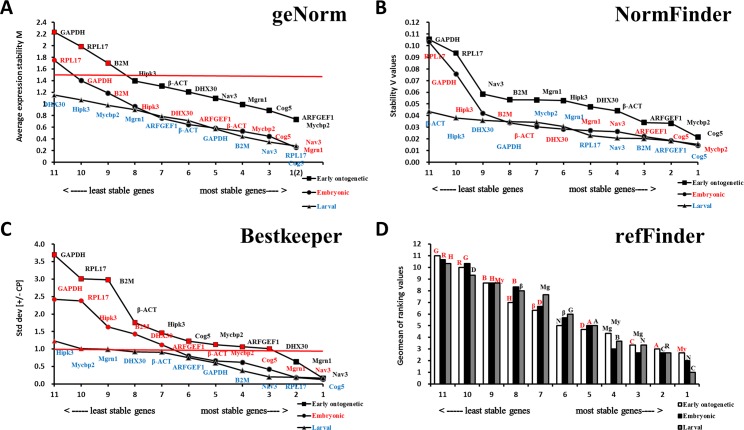
Determination of the expression stability during the entire early ontogenetic development stage and in the separate embryonic ontogenesis stage and larval ontogenesis stage according to different programs. (A) geNorm, the red line indicates the geNorm cut-off value of 1.5. (B) NormFinder. (C) BestKeeper, the red line indicates the BestKeeper cut-off value of 1.0. (D) refFinder, the red indicates the gene expression was unstable. G: GAPDH, R: RPL17, H: Hipk3, D: DHX30, A: ARFGEF1, β: β-ACT, N: Nav3, C: Cog5, Mg: Mgrn1, My: Mycbp2, B: B2M.

When the entire early ontogenetic development stage (from two-cell to 32 days after hatching (dah)) was considered, the Cq values of all eleven genes ranged from 18.2 to 36.4 (S1 Table and Fig 1B). Unlike highly expressed *RPL17* (Cq ranging from 18.2 to 29.2) and weakly expressed *Hipk3* (Cq ranging from 29.4 to 35.4), the other candidate reference genes were expressed at moderate levels. Combining the results from geNorm, NormFinder, and BestKeeper programs, *Nav3* and *Mgrn1* were stably expressed genes during the entire early ontogenetic development stage of *E*. *akaara* (Fig 4A, 4B and 4C). The comprehensive ranking of stability according to the integrative refFinder was *Mycbp2*>*ARFGEF1*>*Cog5*>*Mgrn1*>*DHX30*>*Nav3*>*β-ACT*>*Hipk3*>*B2M*>*RPL17*>*GAPDH* (Fig 4D). However, the pairwise variation showed that all V_n_/V_n+1_ were larger than 0.15, which indicated that no optimal number of reference genes was determined (Fig 3A). Therefore, no ideal choice for normalization during the *E*. *akaara* early ontogenetic development stage could be determined, although *Nav3*/*Mgrn1* might be the best option.

When the early ontogenetic development stage was divided into the embryonic ontogenesis stage (from two-cell to newly hatched larvae) and larval ontogenesis stage (from first feeding larvae to 32 dah), consistent with other reports [32, 33], better results were obtained. According to the analysis of geNorm, NormFinder and BestKeeper, *Nav3*, *Mgrn1*, *Cog5*, *Mycbp2*, *β-ACT* and *ARFGEF1* were stably expressed during the embryonic ontogenesis stage, whereas *Cog5*, *RPL17*, *Nav3*, *B2M*, *GAPDH*, *β-ACT*, *ARFGEF1*, *DHX30* and *Mgrn1* were stably expressed during the larval ontogenesis stage (Fig 4A, 4B, and 4C). According to refFinder, the recommended comprehensive ranking of stability was *Nav3*>*Cog5*>*Mgrn1*>*Mycbp2*> *ARFGEF1*>*β-ACT*>*DHX30*>*B2M*>*Hipk3*>*GAPDH*>*RPL17* during the embryonic ontogenesis stage and *Cog5*>*RPL17*>*Nav3*>*B2M*>*ARFGEF1*>*GAPDH*>*Mgrn1*> *β-ACT*>*Mycbp2*>*DHX30*>*Hipk3* during the larval ontogenesis stage (Fig 4D). For the number of genes required, V_3/4_ and V_2/3_ were less than 0.15 during the embryonic ontogenesis stage and larval ontogenesis stage, respectively, suggesting that three and two reference genes were sufficient for normalization, respectively (Fig 3A). Therefore, we recommend *Nav3*/*Cog5*/*Mgrn1* and *RPL17*/*Cog5* as the optimum suitable reference genes for the embryonic ontogenesis stage and the larval ontogenesis stage, respectively.

### Expression stability of candidate reference genes for salinity treatment of *E*. *akaara*

**Fig 5:**
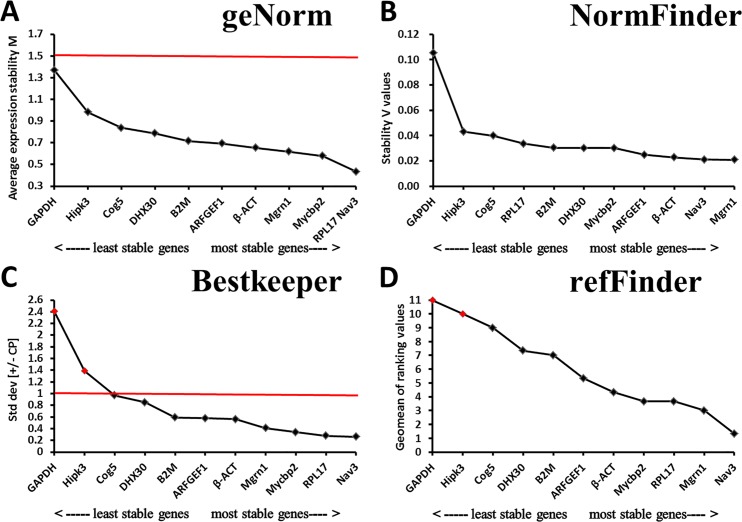
Determination of the expression stability for salinity treatment samples according to different programs. (A) geNorm, the red line indicates the geNorm cut-off value of 1.5. (B) NormFinder. (C) BestKeeper, the red line indicates the BestKeeper cut-off value of 1.0. (D) refFinder, the red indicates the gene expression was unstable.

For the salinity treatment group, the Cq values of the eleven genes ranged between 17.3 and 33.4. *RPL17* and *β-ACT* were the most highly expressed genes, with Cq values ranging from 17.3 to 18.6 and 18.3 to 19.1, respectively, whereas *DHX30* and *Hipk3* were the least expressed, with Cq values ranging from 30.8 to 33.2 and 29.1 to 33.4, respectively (S1 Table and Fig 1C). Because the Cq values of the eleven candidate reference genes between the head kidney and the gills did not differ significantly under salinity treatment, we combined the two tissues to evaluate the gene expression stability. The integrative analysis of the geNorm, NormFinder, and BestKeeper programs showed that most of the candidate reference genes were stable, except for *Hipk3* and *GAPDH* (Fig 5A, 5B and 5C). The refFinder recommended ranking of stability was *Nav3*>*Mgrn1*>*RPL17*>*Mycbp2*>*β-ACT*>*ARFGEF1*>*B2M*>*DHX30*>*Cog5*>*Hipk3*>*GAPDH* (Fig 5D). The geNorm analysis showed that the V_3/4_ value was less than 0.15; therefore, three reference genes were required (Fig 3A). In conclusion, we recommend *Nav3*/*Mgrn1*/ *RPL17* as the most suitable reference genes for *E*. *akaara* under salinity treatment.

### Expression stability of candidate reference genes for *Vibrio alginolyticus* challenged *E*. *akaara*

**Fig 6:**
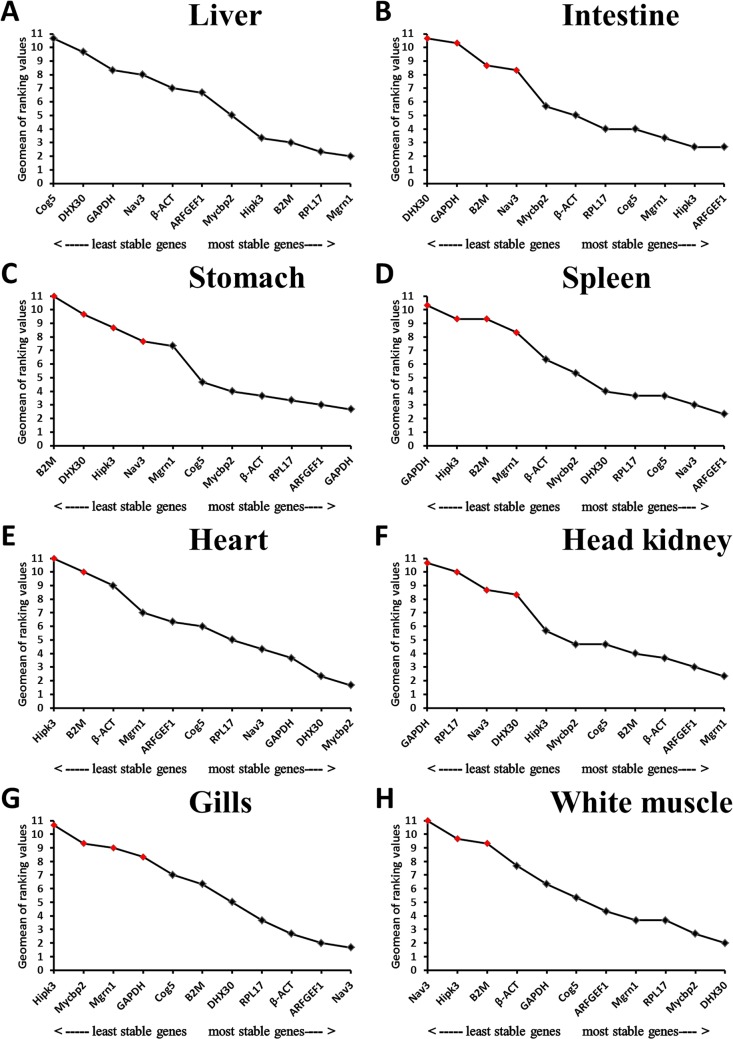
Geomean of ranking values of the 11 candidate reference genes calculated by refFinder for *V*. *alginolyticus* challenged *E*. *akaara*. The red diamond indicates the gene expression was unstable as determined by geNorm, NormFinder, or BestKeeper. Ranking of the gene expression stability was performed for the (A) liver, (B) intestine, (C) stomach, (D) spleen, (E) heart, (F) head kidney, (G) gills, and (H) white muscle.

As shown in S1 Table and Fig 1D, the Cq values of the eleven genes ranged between 17.2 and 36.5. The most highly expressed gene was *RPL17* in the liver, intestine, stomach and spleen; *β-ACT* in the head kidney, heart and gills; and *GAPDH* in white muscle. The least expressed gene was *Mgrn1* in the liver, intestine, spleen, heart, head kidney, gills and white muscle and *DHX30* in the stomach. For the optimal number of genes required for data normalization, the V_2/3_ values were lower than 0.15 in the intestine, heart, and gills, whereas the V_3/4_ values were lower than 0.15 in the liver, stomach, spleen, head kidney, and white muscle; therefore, two and three reference genes were required for normalization, respectively (Fig 3B). The detailed stability ranking according to the integrative refFinder is shown in Fig 6. Based on these results, the most suitable reference genes for *E*. *akaara* were *RPL17*/*Mgrn1*/*B2M* in liver, *ARFGEF1*/*Hipk3* in intestine, *GAPDH*/*ARFGEF1*/*RPL17* in stomach, *ARFGEF1*/*Nav3*/*Cog5* in spleen, *Mycbp2*/*DHX30* in heart, *Mgrn1*/*ARFGEF1*/*β-ACT* in head kidney, *ARFGEF1*/*Nav3* in gills, and *DHX30*/*Mycbp2*/*RPL17* in white muscle,. Although more genes were stably expressed in each tissue, the genes that could be universally used in all the tested tissues were *ARFGEF1*, *β-ACT* and *Cog5*. Considering the complexity and susceptibility of the experimental studies and the V values for each tissue, three reference genes were most appropriate for the normalization of different tissues following exposure to a pathogen. Therefore, we recommend *ARFGEF1*/*β-ACT*/*Cog5* as the most suitable reference genes for all the tested tissues of *E*. *akaara* challenged by *V*. *alginolyticus*.

### The universality of candidate reference genes for *E*. *akaara* in all tested conditions

**Fig 7:**
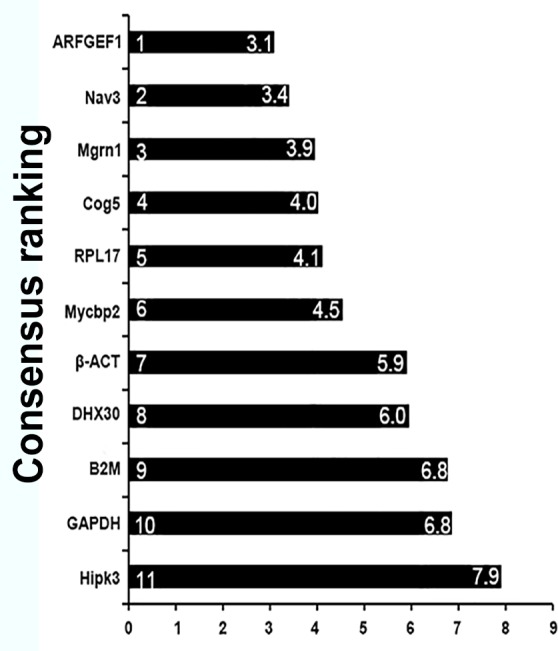
Consensus ranking of the 11 candidate reference genes.

A universally used reference gene would be popular because of its convenience. To find a reference gene that could be universally applied for *E*. *akaara*, we listed the stability status and evaluated the consensus ranking performance of eleven candidate reference genes for all tested conditions. In Table 2, the candidate reference genes are divided into two groups: one group with stable gene expression, and one group with unstable expression. The stably expressed genes were screened according to geNorm, NormFinder and BestKeeper, which must simultaneously satisfy the stability thresholds of these programs. The consensus ranking of each gene was calculated from the geometric mean of the refFinder ranking under all tested conditions. As shown in Fig 7, *ARFGEF1*, *Nav3*, *Mgrn1*, and *Cog5* were the top four genes, whereas in Table 2, only *Cog5* and *ARFGEF1* were listed in the column of stably expressed genes among all the experimental conditions of *E*. *akaara*. Therefore, we recommend *Cog5* and *ARFGEF1* as the optimal choices for universal reference genes in *E*. *akaara*.

**Table 2:** Expression stability ranking of the 11 candidate reference genes.

**Condition**	**Stable expressed gene**	**Unstable expressed gene**
Gonad development phase	**Cog5** Nav3 **ARFGEF1** RPL17 Mycbp2 Mgrn1 B2M	GAPDH DHX30 Hipk3 β-ACT
Embryonic ontogenesis stage	Nav3 **Cog5** Mgrn1 Mycbp2 **ARFGEF1** β-ACT	DHX30 B2M Hipk3 RPL17 GAPDH
Larval ontogenesis stage	**Cog5** RPL17 Nav3 B2M **ARFGEF1** GAPDH β-ACT Mgrn1 DHX30	Mycbp2 Hipk3
Salinity treatment	Nav3 Mgrn1 Mycbp2 RPL17 β-ACT **ARFGEF1** B2M DHX30 **Cog5**	Hipk3 GAPDH
*Vibrio alginolyticus* challenge		
Liver	Mgrn1 RPL17 B2M Hipk3 Mycbp2 **ARFGEF1** β-ACT Nav3 GAPDH DHX30 **Cog5**	
Intestine	**ARFGEF1** Hipk3 Mgrn1 **Cog5** RPL17 β-ACT Mycbp2	Nav3 B2M GAPDH DHX30
Stomach	GAPDH **ARFGEF1** RPL17 β-ACT Mycbp2 **Cog5** Mgrn1	Nav3 Hipk3 DHX30 B2M
Spleen	**ARFGEF1** Nav3 **Cog5** RPL17 DHX30 Mycbp2 β-ACT	Mgrn1 B2M Hipk3 GAPDH
Heart	Mycbp2 DHX30 GAPDH Nav3 RPL17 **Cog5 ARFGEF1** Mgrn1 β-ACT	B2M Hipk3
Head kidney	Mgrn1 **ARFGEF1** β-ACT B2M **Cog5** Mycbp2 Hipk3	DHX30 Nav3 RPL17 GAPDH
Gills	Nav3 **ARFGEF1** β-ACT RPL17 DHX30 B2M **Cog5**	GAPDH Mgrn1 Mycbp2 Hipk3
White muscle	DHX30 Mycbp2 RPL17 Mgrn1 **ARFGEF1 Cog5** GAPDH β-ACT	B2M Hipk3 Nav3

### Validation of the recommended reference genes

**Fig 8:**
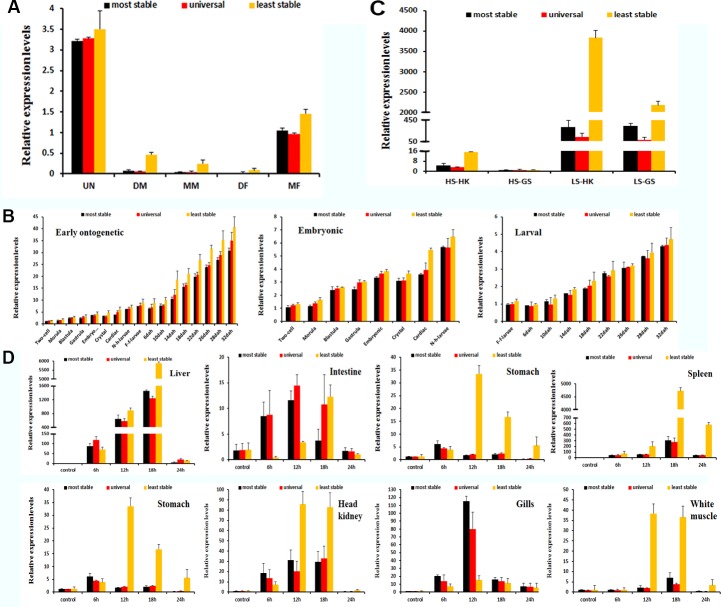
Validation of the recommended reference genes. Expression profiles of *MSTN* and *IL-1β* were analysed using the most stable, the recommended universal and the least stable reference genes during different gonad development phases (A), during the early ontogenetic development stage (B), for the salinity treatment of *E*. *akaara* (C), and for *V*. *alginolyticus* challenged *E*. *akaara* (D). Bars represent the means and standard error of three biological replications. HS: high salinity, LS: low salinity, HK: head kidney, GS: gills.

To examine the availability of the identified most stable reference genes, recommended universal reference genes, and the least stable reference genes, two extensively studied genes, *MSTN* and *IL-1β*, were used as the target genes in different experimental conditions. The relative quantification of these two genes varied according to the different reference genes used during normalization (Fig 8).

Comparing the relative expression levels of *MSTN* and *IL-1β* revealed expression differences of the target genes when normalized with the most stable, the recommended universal and the least stable reference genes. Compared with the least stable genes, the most stable genes and the recommended universal genes showed clearly similar levels of gene expression. Therefore, the recommended universal genes could be used for normalization of target genes under all experimental conditions.

## Discussion

Normalization is essential to obtain accurate and reliable quantitative gene expression data from RT-qPCR analyses. Therefore, the systematic validation of reference genes for different experimental conditions is necessary [34]. During the past few years, the number of articles reporting the validation of reference genes in fishes has increased [35–40], but most studies explore the stability of traditional reference genes such as *β-ACT*, *GAPDH*, *B2M* and *EF1*α. However, transcriptome-wide surveys of gene expression stability in other species, such as monkey [10], buckwheat [11], and oilseed rape [15], show that many newly identified reference genes are more stably expressed. In this study, large-scale gonadal transcriptome data from *E*. *akaara* were used as the source for reference gene selection. The results of RT-qPCR analysis of the different gonad development phases were consistent with the transcriptome sequencing, and the newly identified candidate reference genes were more stably expressed than the traditional genes in some situations, which demonstrated that transcriptome data are an accurate and useful source for candidate reference gene screening and represented an important strategy for large-scale reference gene selection for non-model organisms.

Many statistical methods are used to determine the stability of gene expression and assess the most suitable reference genes [11, 41, 42]; however, no consensus agreement has emerged on which approach is optimum. To improve the reliability and accuracy of the assessment, the combination of different statistical algorithms is the most reliable method. In this study, geNorm, NormFinder and BestKeeper, the most frequently used statistical programs, were used to assess the expression variation of candidate reference genes. We found that the results generated from these three software applications were very similar, despite some nuances in the stability ranking order that might be caused by the different algorithms. Because of the subtle deviations among software programs, refFinder, which compares and ranks the tested candidate reference genes based on the rankings from each program, was used to integrate the three methods.

To avoid erroneous data that may be obtained using a single reference gene, normalization with multiple reference genes is becoming the most common technique [39, 43]. However, because available reference genes are limited, most previous studies typically apply a single reference gene. For the optimal number of reference genes required, the V value calculated by geNorm can provide a reference. Based on our results, the V_2/3_ or V_3/4_ value was lower than the threshold 0.15 of geNorm under all the different experimental conditions, which indicated that 2 or 3 reference genes were required for normalization. Considering the complexity, susceptibility, economic cost and workload of an experimental study, the number of reference genes for normalization is appropriate and practical, and such a strategy has been widely used in many studies [32].

In previous studies on gene expression using the RT-qPCR method, including investigations on grouper functional genes, *β-ACT*, *GAPDH*, and *B2M* are commonly used as internal controls. However, the stability of expression of these genes in grouper remains unclear. In our study, although *β-ACT* is the most frequently used reference gene in grouper [44–47], this gene was not the most suitable choice in most situations, and different results from other studies have made this gene a controversial reference gene. *β-ACT* was a stable gene in 12 tissues of the half-smooth tongue sole following LPS or bacterial challenge [48], but the expression varied to different extents in turbot tissues before and after bacterial challenge [49], during Kuruma shrimp growth and developmental stages [50], and under environmental stresses in *Ciona savignyi* [32]. The different performances of *β-ACT* may be due to its multiple functions not only as a ubiquitous cytoskeleton protein involved in cell structure and motility [1] but also because it participates in diverse biological processes such as transcription and stress response [51]. Although *GAPDH* and *B2M* were identified as stable reference genes by the gonadal transcriptome data, these genes were unusable under certain situations in the present study. For example, *GAPDH* was identified but could not be used in the gonad development phase, embryonic ontogenesis stage, for the salinity treatment, and in many tissues after *V*. *alginolyticus* challenge, and *B2M* could not be used in the embryonic ontogenesis stage and most tissues after *V*. *alginolyticus* challenge. The instability of *GAPDH* has been reported in numerous other systems, including different developmental stages of Atlantic halibut [35], embryonic development in zebra fish [38], virus-infected salmon [52], and different tissue types after bacterial infection in Japanese flounder [39]. The performance of *GAPDH* may be due to its diverse range of functions in the glycolysis cycle, nuclear RNA export, DNA replication and repair, protein phosphotransferase/kinase reactions, membrane transport and fusion, translational regulation and phosphotransferase activity [6], making the expression sensitive to many perturbations in cellular homeostasis [53]. *B2M* was identified as the least stable gene across all tested tissue types after *V*. *alginolyticus* challenge. Consistent with our results, several studies report considerable variation of *B2M* across tissues, including in turbot and flounder when infected by megalocytivirus and *Edwardsiella tarda* [39, 43, 49]. The instability of *B2M* was induced by infection, which may reflect its role in antigen binding and presentation by the major histocompatibility complex (MHC), hence its limitation as a reference gene in such studies [54]. In the current study, none of these three commonly used housekeeping genes showed high stability, indicating that their expression levels were influenced by both the developmental process and the immune response; thus, these housekeeping genes unsuitable as internal controls.

Clearly, a universal reference gene would be more acceptable to researchers because of its convenience. Of the eleven candidate reference genes, based on the integration of the stability results across all tested conditions, only the stability parameters of *Cog5* and *ARFGEF1* satisfied the stability threshold for all the experimental conditions. Although *Cog5* was a newly identified reference gene in fish, previous studies of human non-small cell lung cancer and thyroid cancer used this gene as an internal control [55–57]. Furthermore, in a *Pacific oyster* microarray, *Cog5* was also identified as a candidate reference gene because its mRNA level appeared stable across all analysed tissues [58]. The stability of *Cog5* was closely tied to its function, which is based on the conserved oligomeric Golgi (COG) complex subunit that is involved in the Golgi-associated membrane trafficking and glycoconjugate synthesis [59]. The other gene, ADP-ribosylation factor, *ARFGEF1*, is ubiquitous in eukaryotic cells and functions as a regulator of vesicular traffic and actin remodelling [60]. Previous studies reveal that *ARFGEF1* is one of the most stable reference genes in plants and animals, including wheat, barley, rye, citrus, and beetle [61–64]. Moreover, the combination of *Cog5* and *ARFGEF1* showed similar normalization results with the most stable genes when the relative expression of *MSTN* and *IL-1β* was analysed. Therefore, we recommend that *Cog5* and *ARFGEF1* be used as reference genes for *E*. *akaara* under all tested conditions. However, for a specific experimental condition, the most suitable reference genes are strongly recommended because of the better stability and applicability.

## Conclusions

In summary, the primary conclusions of this study were as follows: (1) the three traditionally used reference genes, *β-ACT*, *GAPDH* and *B2M*, were not applicable for *E*. *akaara* gene expression studies in most situations; (2) the two newly identified reference genes, *Cog5* and *ARFGEF1*, could be universally applied in *E*. *akaara* under all tested conditions; and (3) the most suitable reference genes were identified for each specific experimental conditions of *E*. *akaara*. Additionally, our results might be applicable to other groupers.

## Supporting information

S1 Fig Melting curve analyses of 11 candidate reference genes using RT-qPCR. (TIF)

S2 Fig Expression stability values (M) of the 11 candidate reference genes calculated by geNorm for *V*. *alginolyticus* challenged *E*. *akaara*. Low M values indicate more stable expression. The least stable genes are on the left, and the most stable genes are on the right. The red line indicates the geNorm cut-off value of 1.5. Ranking of the gene expression stability was performed for the (A) liver, (B) intestine, (C) stomach, (D) spleen, (E) heart, (F) head kidney, (G) gills, and (H) white muscle.
(TIF)

S3 Fig Expression stability V values of the 11 candidate reference genes calculated by NormFinder for *V*. *alginolyticus* challenged *E*. *akaara*. Low V values indicate more stable expression. The least stable genes are on the left, and the most stable genes are on the right. Ranking of the gene expression stability was performed for the (A) liver, (B) intestine, (C) stomach, (D) spleen, (E) heart, (F) head kidney, (G) gills, and (H) white muscle.
(TIF)

S4 Fig Expression stability values (SD) of the 11 candidate reference genes calculated by BestKeeper for *V*. *alginolyticus* challenged *E*. *akaara*. Low SD values indicate more stable expression. The least stable genes are on the left, and the most stable genes are on the right. The red line indicates the BestKeeper cut-off value of 1.0. Ranking of the gene expression stability was performed for the (A) liver, (B) intestine, (C) stomach, (D) spleen, (E) heart, (F) head kidney, (G) gills, and (H) white muscle.
(TIF)

S1 File Sample preparation and experimental procedures. (DOCX)

S1 Table Average Cq values ± SD of the 11 candidate reference genes for all the different experimental samples. (DOCX)
