# Supplementary figures and images for: Selection and evaluation of new reference genes for RT-qPCR analysis in Epinephelus akaara based on transcriptome data
Source: PLoS One. 2017 Feb 9;12(2):e0171646. doi: 10.1371/journal.pone.0171646 (PMC5300273; doi:10.1371/journal.pone.0171646)

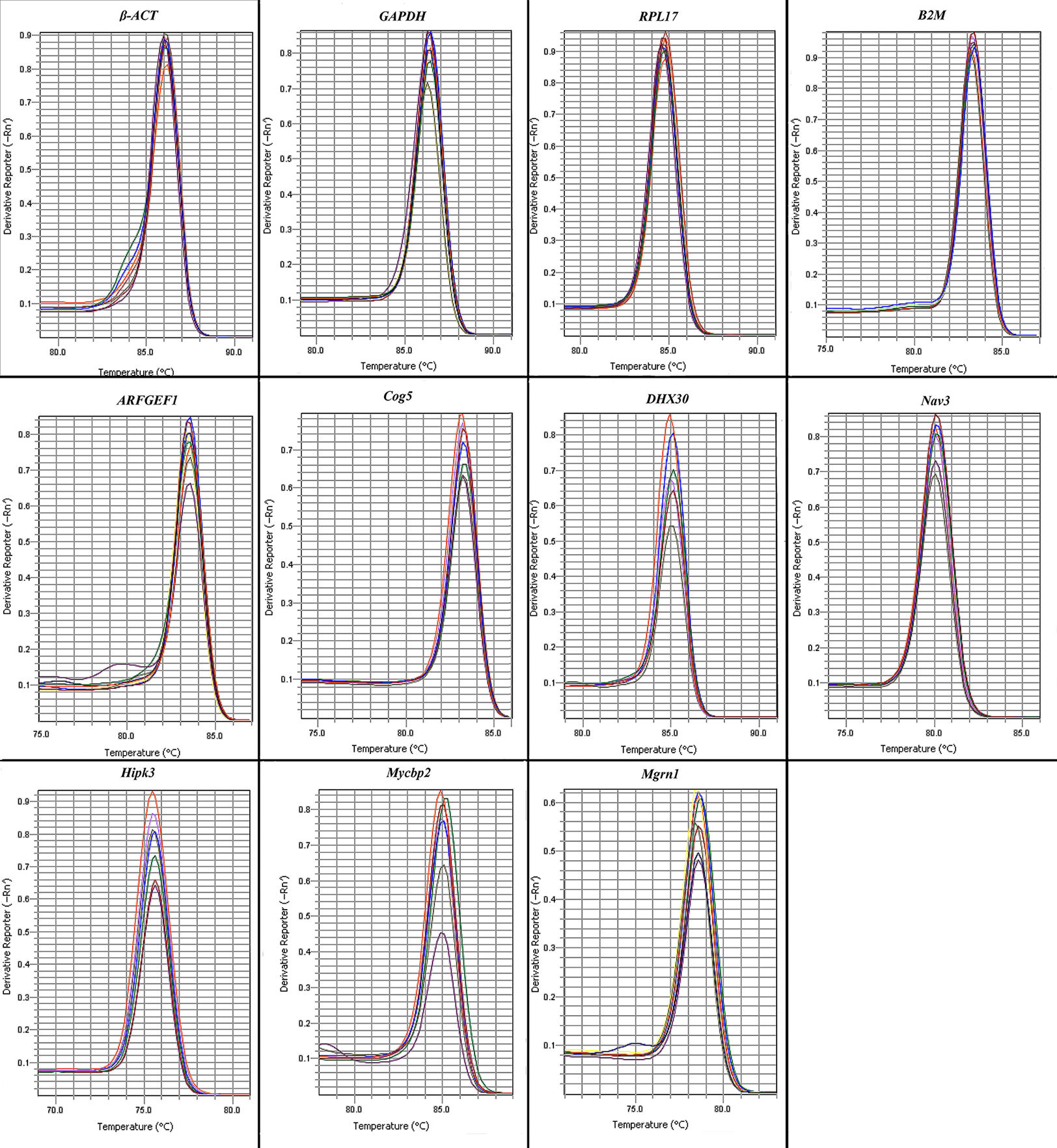

Supplement: S1 Fig — (TIF) [file pone.0171646.s001.tif]

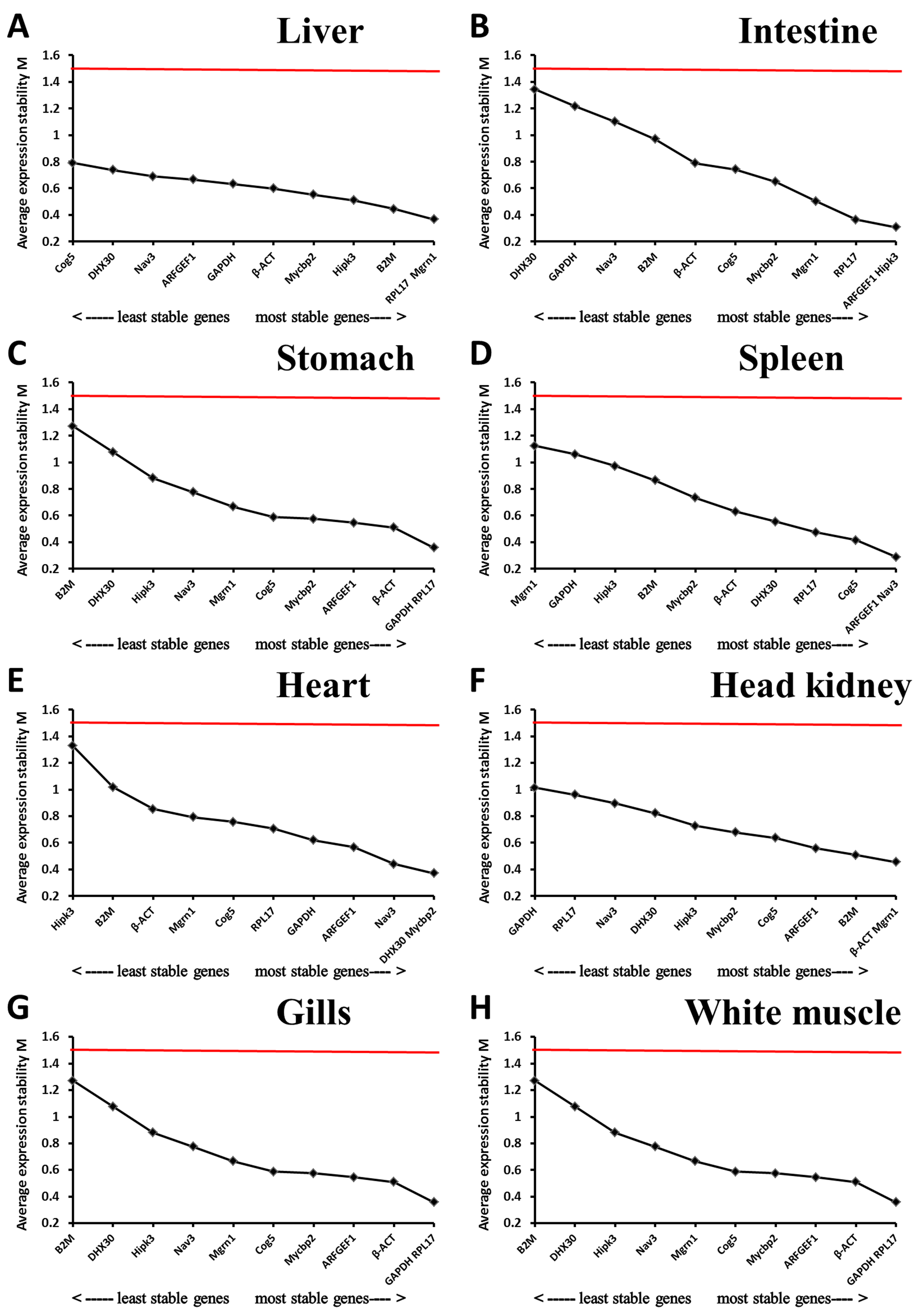

Supplement: S2 Fig — Low M values indicate more stable expression. The least stable genes are on the left, and the most stable genes are on the right. The red line indicates the geNorm cut-off value of 1.5. Ranking of the gene expression stability was performed for the (A) liver, (B) intestine, (C) stomach, (D) spleen, (E) heart, (F) head kidney, (G) gills, and (H) white muscle. (TIF) [file pone.0171646.s002.tif]

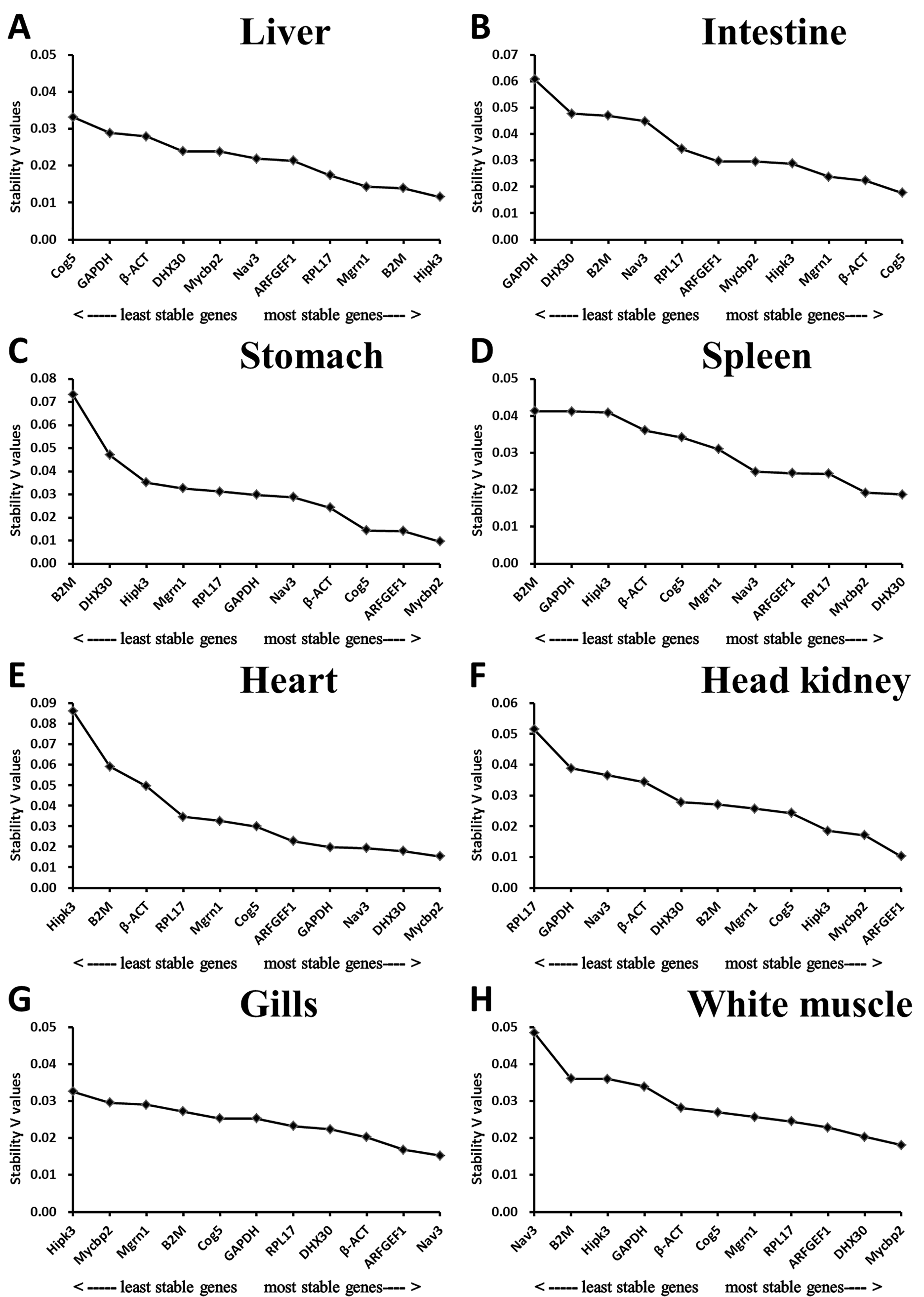

Supplement: S3 Fig — Low V values indicate more stable expression. The least stable genes are on the left, and the most stable genes are on the right. Ranking of the gene expression stability was performed for the (A) liver, (B) intestine, (C) stomach, (D) spleen, (E) heart, (F) head kidney, (G) gills, and (H) white muscle. (TIF) [file pone.0171646.s003.tif]

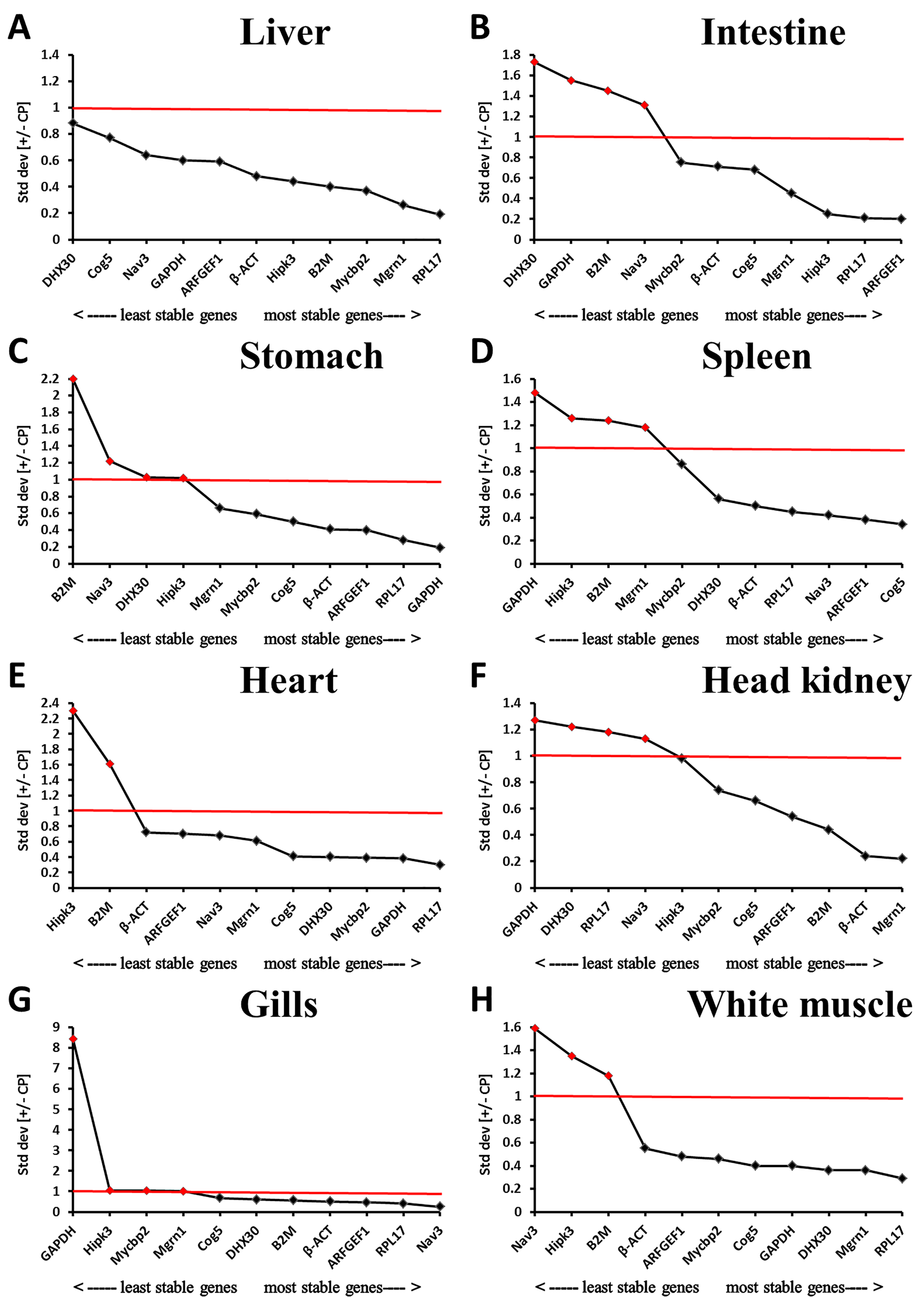

Supplement: S4 Fig — Low SD values indicate more stable expression. The least stable genes are on the left, and the most stable genes are on the right. The red line indicates the BestKeeper cut-off value of 1.0. Ranking of the gene expression stability was performed for the (A) liver, (B) intestine, (C) stomach, (D) spleen, (E) heart, (F) head kidney, (G) gills, and (H) white muscle. (TIF) [file pone.0171646.s004.tif]
