# Supplementary material for: Selection and evaluation of new reference genes for RT-qPCR analysis in Epinephelus akaara based on transcriptome data
Source: PLoS One. 2017 Feb 9;12(2):e0171646. doi: 10.1371/journal.pone.0171646 (PMC5300273; doi:10.1371/journal.pone.0171646)
Supplement: S1 File — (DOCX) [file pone.0171646.s005.docx]

# Sample preparation and experimental procedures

## Ethics statement

This study was conducted in strict accordance with the guidelines for the Care and Use of Laboratory Animals. The Institutional Animal Care and Use Committee of Xiamen University approved the protocol. All fish surgery procedures were conducted under MS-222 (Tricaine Methanesulfonate) to induce sedation and anaesthesia.

## Detailed information on sample preparation

For the different developmental stages of gonads, fish were collected from December 2012 to June 2014. The estimated ages were between 6 and 96 weeks after hatching (wah). The gonadal developmental stages were identified according to gonadal histology samples at the following five critical stages: undifferentiated-phase (UN), developing female (DF), developing male (DM), mature female (MF) and mature male (MM), which were used for follow-up studies. For each developmental stage, three parallel samples were prepared, and approximately 3 mg of each sample was used for RNA isolation.

For individual development setup, the early ontogenetic developmental stages were determined by observation using a stereoscopic microscope. Samples were collected from two-cell, morula, high blastula, mid-gastrula, embryonic body formation, crystal formation, cardiac differentiation, newly hatched larvae, first feeding larvae, and larvae at 6, 10, 14, 18, 22, 26, 28, and 32 days after hatching (dah) at 28°C. For two-cell to first feeding larvae, 10 embryos or larvae were collected per pool at each time point, whereas for 6 to 32 dah larvae, 1-10 larvae per pool were collected dependent on the size and biomass. For each time point, three pooled samples were used.

The fish used for salinity treatment were approximately 8 months old, 130 mm in body length and weighed approximately 29 g. Three individuals from each group were randomly assigned to the high salinity group (40 ‰) and low salinity group (0 ‰). Head kidney and gills, which are two organs that are susceptible to salinity variation, were harvested 20 minutes post-treatment. For each organ, samples of approximately 3 mg were used for RNA isolation.

The *V. alginolyticus* infected individuals were approximately 8 months old and weighed 24 g, with an average body length of 125 mm. They fish were injected intraperitoneally with 0.1 ml of 1 × 10^8^ colony-forming units (CFU)/ml of live *V. alginolyticus*. Fish were sacrificed at 6, 12, 18, and 24 hours post-injection. The control group received an equal volume of sterile saline. Samples from the liver, intestine, stomach, spleen, heart, head kidney, gills and white muscle were collected from three individuals each of the infected and control groups. For each organ, samples of approximately 3 mg were used for RNA isolation.

## RNA isolation and cDNA synthesis

The total RNA from all samples was extracted using RNAiso Plus (TaKaRa, Japan) according to the manufacturer’s instructions. The concentration and purity of the total RNA were determined with a UV-Vis spectrophotometer Q5000 (Quawell, USA). Samples with a concentration greater than 200 ng/μl and A260/280 nm absorbance ratio greater than 1.8 were accepted. The integrity of the total RNA was evaluated using 1% agarose gel electrophoresis. The RNA with clear bands corresponding to 18S and 28S rRNA and the absence of smear was used for cDNA synthesis. A PrimeScript RT reagent Kit with gDNA Eraser (TaKaRa, Japan) was used for the reverse transcription reaction, with the gDNA Eraser to remove genomic DNA. One microgram of total RNA was used for cDNA synthesis according to the manufacturer’s protocol, and the cDNA was stored at -20°C until use.

## Primer design and standard curve analysis

All primer pairs were designed using Primer Premier 5.0 software. To check primer specificity, the products of real-time PCR using cDNA were analysed using 1% agarose gel electrophoresis and Sanger sequencing. For each primer pair, the amplification efficiencies (E) and correlation coefficients (R^2^ values) were determined from the slopes of the standard curves generated using serial dilutions of sample cDNA. The efficiency was calculated as the formula efficiency (%) = ( 10 ^(-1/slope)^ -1 ) ×100. An acceptable efficiency value was between 90% and 110%.

## RT-qPCR Amplification

RT-qPCR was conducted with a QuantStudio^TM^ 6 Flex (Applied Biosystems, USA) using the SYBR Premix DimerEraser (TaKaRa, Japan). For each run, a 20 μl reaction mixture contained 2 μl of template, 10 μl of 2×SYBR Premix DimerEraser, 0.6 μl (10 μM) of each primer, 0.4 μl of ROX and 6.4 μl of dH_2_O. A recommended 3-step PCR standard protocol was followed. The PCR program was 95°C for 30 s, followed by 40 cycles at 95°C for 3 s, 55°C for 30 s and 72°C for 30 s. Melting curve analysis was used to confirm the amplification specificity of each RT-qPCR assay; the primer pairs showed a single, sharp peak indicating that the primers amplified a single, specific PCR product. Each sample was analysed in triplicate, the allowed variation in the Ct values was at ± 0.5 between technical replicates of each duplicate reaction, and the mean Ct values were calculated. Non-template controls (NTC) and non-reverse transcription controls (NRT) were included in each assay to ensure the absence of contamination or primer dimer formation and genomic DNA in the template.
