# Supplementary material for: Selection and evaluation of new reference genes for RT-qPCR analysis in Epinephelus akaara based on transcriptome data
Source: PLoS One. 2017 Feb 9;12(2):e0171646. doi: 10.1371/journal.pone.0171646 (PMC5300273; doi:10.1371/journal.pone.0171646)
Supplement: S1 Table — (DOCX) [file pone.0171646.s006.docx]

| Experiment | Sample | Treatment | β-ACT | GAPDH | RPL17 | B2M | ARFGEF1 | Cog5 | DHX30 | Nav3 | Hipk3 | Mycbp2 | Mgrn1 |
| --- | --- | --- | --- | --- | --- | --- | --- | --- | --- | --- | --- | --- | --- |
| Gonad Development | Gonad | UN | 18.6±0.3 | 19.8±0.6 | 17.5±0.3 | 25.3±0.3 | 26.8±0.3 | 24.6±0.2 | 25.4±0.2 | 22.5±0.3 | 28.0±0.5 | 27.1±0.1 | 26.8±0.2 |
|  |  | DM | 17.9±0.5 | 20.5±0.7 | 17.2±0.2 | 25.0±0.3 | 25.4±0.1 | 24.5±0.1 | 25.6±0.2 | 22.9±0.1 | 28.0±0.6 | 27.5±0.2 | 24.5±0.3 |
|  |  | MM | 18.8±0.1 | 20.1±0.8 | 17.7±0.2 | 24.7±0.3 | 25.2±0.3 | 24.6±0.1 | 25.7±0.2 | 22.5±0.2 | 28.5±0.2 | 25.6±0.3 | 26.6±0.2 |
|  |  | DF | 17.1±0.3 | 17.1±0.7 | 17.6±0.2 | 26.2±0.0 | 25.7±0.3 | 24.5±0.2 | 23.2±0.4 | 22.9±0.0 | 26.6±0.4 | 26.9±0.2 | 23.9±0.3 |
|  |  | MF | 21.4±0.9 | 19.5±0.9 | 17.6±0.3 | 26.0±0.1 | 24.7±0.4 | 24.6±0.1 | 23.3±0.5 | 22.5±0.0 | 26.1±0.1 | 26.9±0.4 | 23.8±0.4 |
| *V.alginolyticus* Challenge | Liver | control | 21.7±0.3 | 21.2±0.1 | 19.3±0.1 | 28.5±0.2 | 28.1±0.3 | 27.3±0.2 | 30.5±0.5 | 28.2±0.1 | 29.5±0.2 | 30.7±0.4 | 31.4±0.3 |
|  |  | 6h | 20.6±0.5 | 19.4±0.4 | 19.6±0.3 | 28.4±0.2 | 27.1±0.5 | 28.4±0.1 | 30.3±0.4 | 28.2±0.2 | 28.5±0.2 | 30.8±0.3 | 31.3±0.3 |
|  |  | 12h | 20.5±0.2 | 20.5±0.1 | 19.3±0.1 | 29.3±0.0 | 28.4±0.3 | 27.3±0.1 | 31.3±0.3 | 29.4±0.3 | 28.7±0.2 | 30.3±0.3 | 31.5±0.2 |
|  |  | 18h | 20.4±0.2 | 20.4±0.5 | 19.3±0.3 | 29.2±0.2 | 28.2±0.3 | 27.6±0.4 | 32.8±0.1 | 29.8±0.1 | 29.2±0.3 | 31.2±0.4 | 31.6±0.4 |
|  |  | 24h | 21.3±0.3 | 21.3±0.3 | 19.6±0.2 | 29.3±0.2 | 29.4±0.3 | 29.6±0.4 | 32.2±0.3 | 29.4±0.1 | 29.6±0.1 | 30.5±0.4 | 31.6±0.3 |
|  | Intestine | control | 20.2±0.2 | 22.2±0.4 | 19.6±0.2 | 25.5±0.0 | 28.2±0.2 | 27.3±0.2 | 31.8±0.1 | 25.5±0.3 | 29.8±0.2 | 29.6±0.4 | 31.4±0.4 |
|  |  | 6h | 19.4±0.3 | 20.8±0.1 | 19.4±0.1 | 25.5±0.4 | 27.9±0.3 | 27.5±0.2 | 27.3±0.0 | 27.6±0.2 | 29.5±0.4 | 28.1±0.2 | 32.2±0.2 |
|  |  | 12h | 20.4±0.3 | 20.3±0.5 | 19.4±0.4 | 27.9±0.2 | 28.0±0.2 | 27.4±0.3 | 29.7±0.3 | 28.4±0.2 | 29.3±0.3 | 27.5±0.3 | 31.5±0.4 |
|  |  | 18h | 22.2±0.2 | 24.6±0.3 | 19.5±0.3 | 29.5±0.3 | 28.1±0.3 | 29.4±0.2 | 33.4±0.2 | 30.8±0.2 | 29.7±0.2 | 29.5±0.4 | 32.5±0.2 |
|  |  | 24h | 20.2±0.2 | 19.3±0.6 | 19.7±0.3 | 27.3±1.0 | 28.2±0.3 | 27.1±0.6 | 31.2±0.4 | 27.4±0.4 | 29.6±0.2 | 28.6±0.3 | 31.4±0.3 |
|  | Stomach | control | 19.7±0.2 | 21.5±0.1 | 19.6±0.5 | 25.3±0.2 | 28.4±0.1 | 27.3±0.2 | 32.4±0.1 | 28.3±0.0 | 30.3±0.1 | 28.6±0.2 | 32.3±0.4 |
|  |  | 6h | 19.5±0.3 | 21.5±0.4 | 19.5±0.2 | 24.3±0.3 | 27.6±0.1 | 26.6±0.0 | 35.7±0.3 | 27.5±0.3 | 28.6±0.4 | 28.4±0.2 | 31.6±0.1 |
|  |  | 12h | 20.3±0.3 | 21.3±0.2 | 19.6±0.3 | 29.5±0.3 | 28.5±0.3 | 27.6±0.5 | 32.4±0.3 | 30.2±0.1 | 31.0±1.0 | 29.8±0.2 | 31.7±0.4 |
|  |  | 18h | 20.5±0.3 | 21.5±0.3 | 19.7±0.3 | 28.7±0.4 | 29.1±0.4 | 27.7±0.1 | 32.5±0.4 | 30.7±0.2 | 30.2±0.2 | 29.7±0.3 | 33.6±0.4 |
|  |  | 24h | 19.8±0.5 | 21.4±0.3 | 19.5±0.4 | 29.8±0.3 | 28.5±0.1 | 28.4±0.3 | 32.6±0.1 | 30.4±0.1 | 32.4±0.3 | 29.6±0.1 | 31.6±0.4 |
|  | Spleen | control | 17.4±0.3 | 26.0±0.3 | 17.5±0.0 | 23.7±0.2 | 28.4±0.2 | 27.3±0.1 | 30.7±0.3 | 25.1±0.2 | 30.3±0.1 | 27.6±0.3 | 32.3±0.4 |
|  |  | 6h | 18.7±0.0 | 26.7±0.2 | 17.5±0.5 | 23.6±0.2 | 28.1±0.3 | 27.5±0.2 | 29.5±0.1 | 24.7±0.3 | 28.3±0.4 | 27.4±0.4 | 33.7±0.2 |
|  |  | 12h | 17.5±0.2 | 28.5±0.4 | 17.4±0.3 | 25.6±0.2 | 28.9±0.1 | 27.5±0.3 | 30.3±0.2 | 25.5±0.3 | 28.5±0.4 | 28.5±0.3 | 33.4±0.3 |
|  |  | 18h | 18.3±0.3 | 30.4±0.2 | 18.7±0.3 | 26.8±0.3 | 28.9±0.2 | 27.3±0.5 | 31.4±0.1 | 25.6±0.2 | 31.8±0.2 | 30.0±0.1 | 36.5±0.4 |
|  |  | 24h | 17.6±0.4 | 29.4±0.3 | 17.2±0.0 | 26.5±0.2 | 27.9±0.2 | 26.5±0.3 | 30.6±0.4 | 24.5±0.1 | 30.8±0.1 | 29.2±0.3 | 32.5±0.4 |
|  | Heart | control | 18.5±0.4 | 26.3±0.2 | 19.5±0.2 | 23.2±0.1 | 27.0±0.1 | 27.5±0.2 | 27.3±0.1 | 25.3±0.2 | 29.7±0.3 | 27.5±0.2 | 30.1±0.2 |
|  |  | 6h | 20.4±0.2 | 25.4±0.2 | 19.4±0.0 | 24.2±0.1 | 26.4±0.3 | 27.5±0.4 | 28.5±0.1 | 26.5±0.0 | 24.6±0.2 | 28.3±0.3 | 31.4±0.3 |
|  |  | 12h | 19.4±0.4 | 26.5±0.3 | 19.3±0.3 | 27.4±0.3 | 28.3±0.2 | 26.8±0.1 | 28.2±0.1 | 27.2±0.2 | 28.7±0.2 | 28.6±0.4 | 30.6±0.4 |
|  |  | 18h | 18.5±0.3 | 26.3±0.3 | 19.4±0.3 | 26.3±0.3 | 27.9±0.2 | 26.4±0.2 | 28.5±0.2 | 27.1±0.1 | 25.6±0.4 | 28.5±0.1 | 31.4±0.3 |
|  |  | 24h | 18.1±0.1 | 26.6±0.3 | 18.6±0.3 | 27.5±0.4 | 28.2±0.4 | 27.4±0.3 | 28.7±0.2 | 27.4±0.2 | 31.3±0.2 | 28.6±0.3 | 30.0±0.1 |
|  | Head kidney | control | 17.6±0.1 | 26.0±0.2 | 19.6±0.2 | 27.4±0.2 | 27.6±0.3 | 27.5±0.2 | 30.5±0.1 | 25.9±0.1 | 29.1±0.1 | 27.6±0.2 | 32.3±0.4 |
|  |  | 6h | 17.4±0.3 | 24.5±0.2 | 18.1±0.1 | 27.4±0.2 | 27.0±0.4 | 26.6±0.1 | 28.6±0.4 | 25.6±0.4 | 28.5±0.4 | 27.9±0.1 | 32.2±0.3 |
|  |  | 12h | 17.5±0.1 | 27.5±0.2 | 17.6±0.2 | 27.6±0.4 | 27.3±0.2 | 26.2±0.1 | 29.2±0.4 | 27.4±0.2 | 28.3±0.1 | 27.4±0.4 | 32.5±0.1 |
|  |  | 18h | 17.5±0.4 | 28.5±0.4 | 21.2±0.2 | 27.8±0.4 | 28.6±0.0 | 28.0±0.5 | 32.4±0.4 | 29.1±0.7 | 30.6±0.0 | 29.6±0.0 | 32.4±0.1 |
|  |  | 24h | 17.4±0.5 | 27.7±0.0 | 18.2±0.4 | 28.7±0.3 | 28.2±0.2 | 27.2±0.7 | 31.3±0.2 | 27.8±0.8 | 30.8±0.2 | 28.8±0.6 | 32.8±0.1 |
|  | Gills | control | 17.7±0.3 | 29.1±0.1 | 19.2±0.1 | 26.2±0.2 | 26.3±0.4 | 27.7±0.1 | 28.6±0.3 | 27.3±0.2 | 29.3±0.1 | 27.1±0.1 | 33.0±0.4 |
|  |  | 6h | 17.8±0.0 | 29.8±0.3 | 18.2±0.2 | 26.3±0.3 | 26.1±0.2 | 25.7±0.3 | 29.6±0.3 | 27.1±0.0 | 27.6±0.2 | 26.6±0.3 | 32.1±0.5 |
|  |  | 12h | 18.7±0.0 | 30.9±0.3 | 18.9±0.3 | 27.2±0.2 | 26.7±0.4 | 26.0±0.1 | 30.4±0.5 | 27.6±0.1 | 27.0±0.3 | 27.6±0.4 | 34.4±0.2 |
|  |  | 18h | 19.0±0.7 | 31.9±0.3 | 19.4±0.3 | 26.2±0.2 | 27.0±0.3 | 27.3±0.3 | 31.4±0.3 | 27.7±0.3 | 29.1±0.9 | 28.9±0.3 | 35.2±0.3 |
|  |  | 24h | 19.0±0.4 | 31.8±0.7 | 18.6±0.2 | 27.7±0.3 | 27.4±0.6 | 26.8±0.2 | 30.8±0.2 | 27.5±0.4 | 29.8±0.1 | 29.7±0.8 | 32.9±0.3 |
|  | White muscle | control | 21.9±0.2 | 19.1±0.5 | 20.8±0.1 | 28.3±0.3 | 29.3±0.3 | 29.3±0.1 | 31.5±0.4 | 27.6±0.1 | 29.8±0.2 | 29.5±0.3 | 34.4±0.2 |
|  |  | 6h | 23.3±0.2 | 17.7±0.3 | 20.5±0.4 | 30.7±0.4 | 28.3±0.1 | 29.7±0.2 | 31.7±0.3 | 27.9±0.3 | 28.3±0.1 | 29.7±0.2 | 34.3±0.3 |
|  |  | 12h | 22.7±0.3 | 18.4±0.2 | 20.4±0.2 | 32.2±0.2 | 29.6±0.3 | 29.6±0.4 | 32.0±0.1 | 32.3±0.1 | 31.2±0.1 | 30.6±0.4 | 34.5±0.4 |
|  |  | 18h | 21.7±0.3 | 18.1±0.1 | 20.3±0.2 | 30.8±0.2 | 29.3±0.3 | 28.8±0.4 | 32.5±0.1 | 30.6±0.2 | 29.6±0.2 | 30.6±0.2 | 34.4±0.3 |
|  |  | 24h | 22.8±0.2 | 18.4±0.2 | 21.1±0.1 | 32.5±0.5 | 28.5±0.4 | 28.6±0.4 | 31.5±0.2 | 30.3±0.4 | 33.0±0.2 | 30.1±0.4 | 33.5±0.2 |
| Early  Ontogenetic Development Stage | All | Two-cell | 22.1±0.4 | 24.2±0.3 | 27.0±0.1 | 36.4±0.3 | 29.7±0.1 | 29.6±0.1 | 31.2±0.1 | 27.8±0.1 | 30.9±0.1 | 31.0±0.1 | 30.4±0.3 |
|  |  | Morula | 23.1±0.2 | 26.0±0.2 | 29.2±0.3 | 34.5±0.1 | 30.5±0.1 | 30.0±0.0 | 31.0±0.2 | 27.5±0.2 | 29.4±0.2 | 31.3±0.2 | 30.4±0.2 |
|  |  | High blastula | 27.1±0.3 | 29.0±0.5 | 24.7±0.2 | 34.6±0.1 | 32.3±0.2 | 30.5±0.4 | 32.6±0.5 | 27.6±0.2 | 33.7±0.4 | 32.5±0.1 | 30.4±0.3 |
|  |  | Mid-gastrula | 24.1±0.2 | 28.6±0.4 | 22.0±0.3 | 31.4±0.3 | 31.4±0.3 | 30.3±0.4 | 31.6±0.2 | 27.5±0.1 | 32.4±0.3 | 32.1±0.3 | 30.6±0.4 |
|  |  | Embryonic body formation | 25.1±0.5 | 31.3±0.4 | 22.8±0.4 | 34.5±0.2 | 31.3±0.3 | 30.6±0.2 | 33.5±0.1 | 27.6±0.2 | 33.3±0.2 | 32.3±0.3 | 30.6±0.1 |
|  |  | Crystal formation | 24.8±0.2 | 30.0±0.2 | 22.3±0.2 | 34.3±0.2 | 31.4±0.3 | 30.7±0.3 | 33.5±0.2 | 27.8±0.1 | 34.9±0.2 | 32.3±0.1 | 30.7±0.0 |
|  |  | Cardiac differentiation | 25.0±0.3 | 29.8±0.2 | 22.7±0.0 | 34.7±0.2 | 31.2±0.3 | 30.4±0.5 | 34.4±0.4 | 27.7±0.0 | 35.4±0.3 | 32.7±0.2 | 30.5±0.3 |
|  |  | Newly hatched larvae | 21.1±0.4 | 24.8±0.4 | 19.8±0.3 | 30.7±0.3 | 29.4±0.5 | 29.5±0.3 | 31.4±0.3 | 27.8±0.0 | 31.5±0.4 | 30.8±0.1 | 30.5±0.2 |
|  |  | First feeding larvae | 21.5±0.5 | 22.3±0.4 | 18.6±0.1 | 27.6±0.2 | 28.9±0.1 | 27.8±0.0 | 31.5±0.4 | 27.6±0.3 | 30.3±0.2 | 27.7±0.4 | 28.1±0.0 |
|  |  | 6dah | 19.4±0.1 | 20.3±0.1 | 18.2±0.1 | 27.2±0.1 | 28.2±0.1 | 27.7±0.2 | 32.5±0.1 | 28.0±0.0 | 31.6±0.1 | 28.5±0.1 | 28.4±0.1 |
|  |  | 10dah | 20.0±0.0 | 20.3±0.1 | 18.7±0.1 | 27.7±0.1 | 27.8±0.1 | 28.0±0.2 | 32.7±0.1 | 27.6±0.0 | 31.7±0.4 | 28.2±0.1 | 28.3±0.1 |
|  |  | 14dah | 19.2±0.2 | 20.7±0.1 | 18.6±0.1 | 28.5±0.4 | 28.2±0.0 | 27.3±0.3 | 32.9±0.0 | 27.8±0.0 | 31.6±0.2 | 29.8±0.1 | 29.4±0.2 |
|  |  | 18dah | 19.2±0.3 | 20.6±0.1 | 18.6±0.1 | 27.9±0.4 | 29.3±0.2 | 27.8±0.1 | 32.1±0.1 | 27.6±0.3 | 30.7±0.3 | 30.0±0.1 | 29.5±0.2 |
|  |  | 22dah | 21.4±0.1 | 21.8±0.2 | 18.7±0.3 | 28.7±0.2 | 30.5±0.1 | 28.0±0.0 | 29.7±0.3 | 27.6±0.3 | 31.6±0.1 | 31.4±0.2 | 30.7±0.2 |
|  |  | 26dah | 19.7±0.1 | 20.5±0.3 | 18.2±0.1 | 28.2±0.4 | 29.0±0.0 | 27.8±0.1 | 30.2±0.3 | 27.8±0.1 | 32.5±0.1 | 30.5±0.1 | 30.6±0.1 |
|  |  | 28dah | 20.1±0.2 | 20.5±0.3 | 18.6±0.3 | 27.7±0.3 | 29.5±0.2 | 27.7±0.2 | 31.4±0.0 | 27.7±0.1 | 33.6±0.3 | 29.6±0.1 | 30.6±0.3 |
|  |  | 32dah | 21.5±0.1 | 20.6±0.4 | 18.4±0.2 | 28.2±0.4 | 31.0±0.1 | 27.7±0.4 | 32.4±0.0 | 27.3±0.2 | 34.6±0.2 | 31.6±0.2 | 31.7±0.2 |
| Salinity Treatment | Head kidney | High salinity | 19.1±0.1 | 26.3±0.0 | 18.3±0.4 | 25.0±0.7 | 28.9±0.2 | 28.6±0.3 | 33.2±0.2 | 25.2±0.2 | 33.4±0.2 | 29.0±0.4 | 32.2±0.2 |
|  |  | Low salinity | 18.3±0.3 | 23.3±0.3 | 18.3±0.5 | 24.2±0.6 | 28.5±0.4 | 28.3±0.2 | 33.0±0.1 | 25.2±0.4 | 30.1±0.2 | 29.7±0.7 | 30.3±1.0 |
|  | Gills | High salinity | 18.6±0.1 | 29.0±0.4 | 17.3±0.1 | 23.8±0.3 | 27.3±0.4 | 25.8±0.1 | 30.8±0.4 | 25.1±0.3 | 29.1±0.3 | 29.2±0.5 | 30.4±0.1 |
|  |  | Low salinity | 19.1±0.1 | 30.2±0.6 | 18.6±0.3 | 24.1±0.9 | 28.1±0.6 | 27.5±0.3 | 32.5±0.4 | 25.6±0.4 | 29.7±0.1 | 29.4±0.3 | 31.0±0.6 |
